# Fatal interstitial lung disease caused by Panitumumab-containing chemotherapy regimen

**DOI:** 10.3332/ecancer.2018.841

**Published:** 2018-06-07

**Authors:** Osamah Al-asadi, Manar Almusarhed, Syed Azhar J Rizvi, Wasiru Saka

**Affiliations:** 1 Department of Oncology, Milton Keynes University Hospital, NHS Foundation Trust, Standing Way, MK65LD, UK; 2 School of Medicine, University of Buckingham, Buckingham MK18 1EG, UK; 3 University of Babylon, Hillah 51002, Iraq

**Keywords:** colon cancer, unwanted effects/adverse reactions, pharmacology and therapeutics, interstitial lung disease

## Abstract

Fatal interstitial lung disease (ILD) is one of the rare side effects of Panitumumab. Both fatal and non-fatal ILD have been reported mainly in the Japanese population. We report a case of a nonsmoking Caucasian man with the diagnosis of metastatic rectal cancer (K-RAS wild-type) who developed fatal ILD after receiving a Panitumumab-containing chemotherapy regimen. He initially presented with a locally advanced rectal cancer (T3N2M0) for which he received neoadjuvant chemoradiotherapy. Before the rectal surgery, he was found to have liver metastases which were considered potentially resectable. The decision was to delay the rectal surgery and to start systemic treatment. He was started on Modified De Gramont regimen (folinic acid and fluorouracil) plus Oxaliplatin with Panitumumab. Six months later, he underwent rectal surgery which showed a complete response. He continued on systemic treatment while awaiting liver metastasectomy. After three courses of Modified De Gramont plus Oxaliplatin with Panitumumab, he was admitted with few days’ history of increasing shortness of breath. High-resolution computed tomography of the lungs showed the features of interstitial pneumonitis. Despite receiving appropriate treatment, he continued to deteriorate and died due to respiratory failure.

## Background

Panitumumab is licensed for use in metastatic colorectal cancer in conjunction with chemotherapy after reported benefit in phase III randomised trial in the metastatic setting (PRIME) [1]. However, data are lacking about the incidence of Panitumumab-related interstitial lung disease (ILD).

Both the fatal and nonfatal ILDs have been reported mainly in the Japanese population where Panitumumab was approved in 2010 [2]. Here, we report a case of Panitumumab-induced fatal ILD.

## Case presentation

A 62-year-old Caucasian man, nonsmoker, had recto-sigmoid carcinoma in September 2015. Initial investigations showed the cancer was T3, N2 and M0 (grade II adenocarcinoma). He received neoadjuvant chemoradiotherapy with Capecitabine and radiotherapy (50Gy in 25 fractions) which was completed in December 2015.

**Figure 1.**
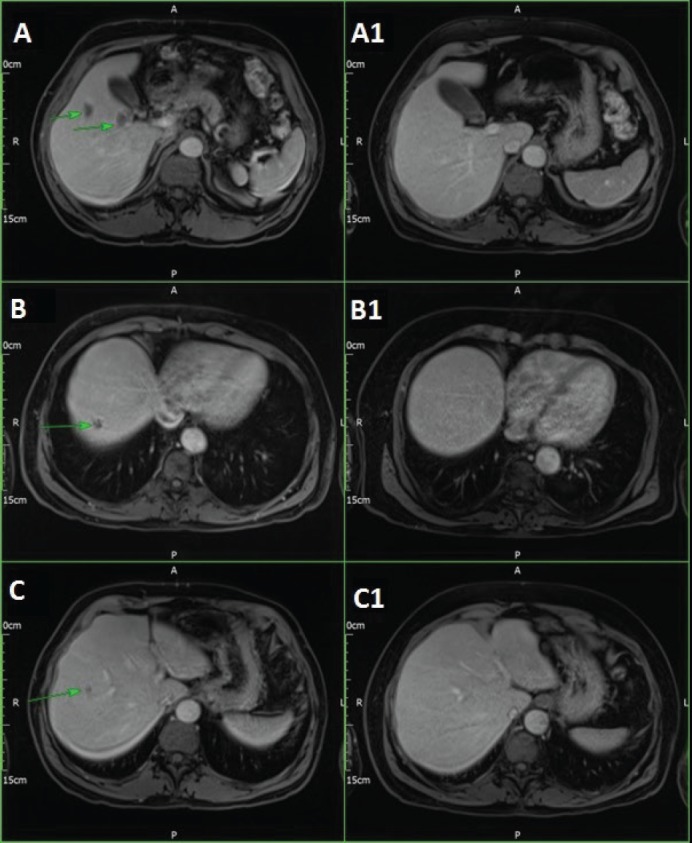
Panitumumab-induced fatal ILD. MRI liver, March 2016 (A, B, C) and September 2016 (A1, BA, C1).

Post chemo-radiotherapy, computed tomography (CT) scan and positron emission tomography (PET)/CT showed favourable rectal tumour response, but there were six small new liver lesions on magnetic resonance imaging (MRI). He was started on Modified De Gramont regimen plus Oxaliplatin in March 2016, Panitumumab was added in May 2016 as K-RAS was wild-type. Reassessment CT scan, MRI and PET scans in September 2016 showed a very good response in the liver and rectal primary lesions with no evidence of extra-hepatic disease. Only two lesions had been identified in the liver MRI while the rest of the lesions disappeared (Figure 1). He underwent resection of the rectal disease with loop ileostomy by end of January 2017. The postoperative pathology showed a complete response. After recovery from bowel surgery, he continued on systemic chemotherapy and Panitumumab early March 2017 for three more cycles.

In May 2017, he was admitted with a 3-day history of fever, dry cough, progressive shortness of breath and decreased exercise tolerance. SpO2 was reduced at 88%. The total number of Panitumumab treatment cycles the patient received prior to this acute admission was 18.

## Investigations

**Figure 2.**
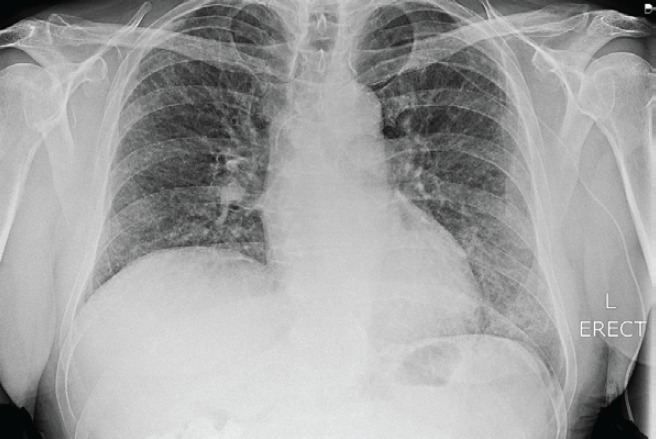
Panitumumab-induced fatal ILD. Chest X-ray on day 1 of admission, showing mild interstitial shadowing in both lungs.

**Figure 3.**
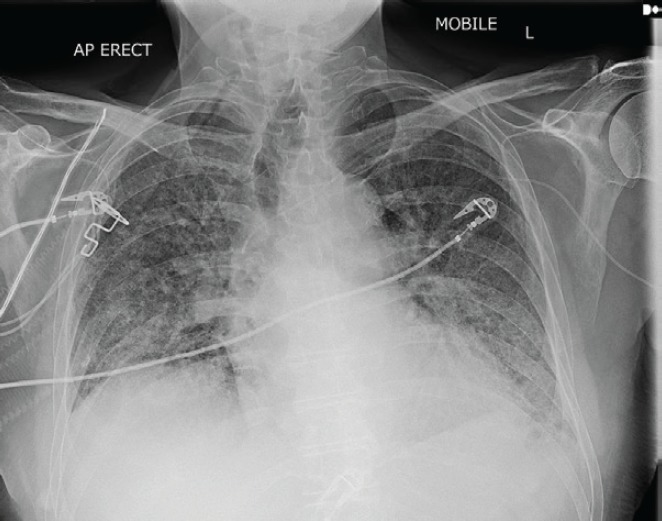
Panitumumab-induced fatal ILD. Chest X-ray on day 17 of admission, showing further deterioration.

**Figure 4.**
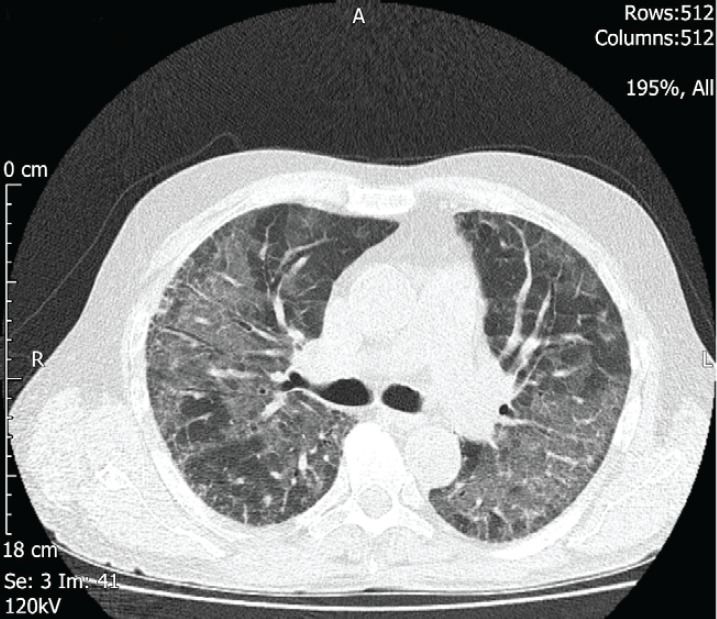
Panitumumab-induced fatal ILD. HRCT of the chest, axial image, two days after the admission, showing bilateral patchy ground glass changes.

Serial chest X-rays (Figures 2 and 3) showed diffuse reticulonodular shadowing. High-resolution computed tomography (HRCT) (Figure 4) showed nonspecific interstitial pneumonia.

Septic screen including blood culture, sputum culture, urine culture, human immunodeficiency virus (HIV) test, methicillin-resistant staphylococcus aureus (MRSA) polymerase chain reaction (PCR) and Pneumocystis Jirovecii PCR were all negative. In addition, autoimmune disorders were also excluded as antinuclear antibodies (ANA), antineutrophil cytoplasmic antibodies (ANCA) and rheumatoid factor (RF) were negative. Unfortunately, the patient was clinically unfit for bronchoscopy to exclude possible lymphangitis carcinomatosis although it is rare in colorectal cancer and the patient refused further invasive procedures.

Differential diagnosis

1. Pneumocystis jerovicii pneumonia.
2. Lymphangitis carcinomatosis.
3. Pulmonary oedema.
4. Oxaliplatin-induced ILD.

## Treatment

The patient was started on broad-spectrum IV antibiotics and anti-Pneumocystis jirovecii pneumonia treatment was added from day three as per microbiology advice. The HRCT result was discussed with an ILD expert who recommended starting pulse steroid therapy. He also recommended not to consider mechanical ventilation if the patient deteriorates as it would worsen the condition. Methylprednisolone 1g/day was given for 3 days which started on day five after all possible underlying infective causes had been excluded.

The patient’s condition deteriorated and he was transferred to intensive therapy unit (ITU) on day six.

## Outcome and follow-up

In ITU, the patient was treated as type 1 respiratory failure and was started initially on a non-re-breathable mask (15 L/min) to maintain his saturation at about 94%, but due to further desaturation, he was started on transnasal humidified oxygen delivery system (50 L/min). Unfortunately, no improvement was noticed, and the patient died 13 days later.

## Discussion

Panitumumab is a human monoclonal antibody which blocks the activation of the epidermal growth factor receptor (EGFR) resulting in inhibition of signalling that leads to proliferation, angiogenesis and metastasis. This action is achieved with the presence of the wild-type RAS gene in a metastatic colorectal cancer. The US Food and Drug Administration has approved Panitumumab use in combination with Oxaliplatin plus folinic acid and fluorouracil regimen in the first-line treatment of patients with wild-type RAS metastatic colorectal cancer in 2014 based on results of phase 3 clinical trial PRIME [1]. In addition, noninferiority was reported in ASPECCT trial when compared to Cetuximab, the other anti-EGFR monoclonal antibody licensed in the same setting [3]. Pulmonary toxicity is not reported in all metastatic colorectal cancer clinical trials since patients with a history of, or evidence of, interstitial pneumonitis or pulmonary fibrosis were excluded from the clinical studies [2]. A Japanese post-marketing all-case surveillance study from 2010 to 2015, reported Panitumumab-induced ILD incidence of 1.3% and mortality rates of 51.3% [4]. No such data are available in the western population. Early safety studies have found no significant increase in pulmonary complications induced by Oxaliplatin, except for the dyspnoea that may have occurred in the setting of a hypersensitivity reaction [5, 6]. No prospective phase 2 or 3 data are available to our knowledge regarding Oxaliplatin-induced pulmonary toxicity, however, among the few retrospective studies, two studies identified incidence of ILD as 1.5% and 0.64%, respectively [7, 8].

ILD has emerged as a rare but serious adverse event associated with EGFR/tyrosine kinase inhibitors (TKIs). Treatment with various anti-EGFR/TKI agents, including Gefitinib and Erlotinib has been associated with pulmonary toxicity [9]. The aetiology of EGFR associated pulmonary toxicity is not fully understood. However, it can be resulted from impairing the cell and tissue repair sequence as EGFR is expressed on type II pneumocytes, which are involved in alveolar wall repair. Thus, agents targeting the EGFR may impair alveolar repair mechanisms [10, 11]. In addition, preclinical *in vivo* models suggest that the mechanism maybe related to reduced surfactant protein A expression in lung tissues with EGFR inhibition [12]. One meta-analysis evaluated the wide spectrum of the incidence of pulmonary toxicity-related adverse events in non-small cell lung cancer patients treated with Gefitinib [13]. This meta-analysis included 9,054 patients from 23 studies. The findings demonstrated that the overall incidence of ILD was 1.43%. Post-marketing Surveillance of Cetuximab, which is a monoclonal anti-EGFR antibody, in patients with metastatic colorectal cancer reported the incidence of ILDs of 1.2% [14]. Results of a prospective multicentre registry in Japan also reported similar incidence of drug-induced lung injury related to Cetuximab [15].

Mechanical ventilation in ILD is of questionable value. The potential for parenchymal injury caused by high lung stretch that is known as ventilator-induced lung injury has been shown in few clinical studies [16, 17]. The damage is associated with the accumulation of lung neutrophils and cytokines released during the repetitive stretching and closing of alveoli. Moreover, mechanical ventilation could lead to a further decline in respiratory function and reduce the chances of weaning individuals off the ventilator [18, 19]. Many retrospective studies have assessed outcomes in patients with ILD requiring mechanical ventilation due to acute respiratory failure and showed that the majority of patients died while receiving mechanical ventilation or shortly after discharge from the ITU [20–23].

## Conclusions

- Before starting Panitumumab containing therapy, baseline investigations for any possible underlying pulmonary fibrosis or interstitial pneumonitis might be useful. In addition, while on Panitumumab, patients should be monitored regularly for any signs of ILD.
- HRCT should be considered when there is suspicion of ILD like unexplained progressive symptomatic breathlessness or hypoxia.
- Early involvement of respiratory physician is crucial in the management of ILD.
- Although the incidence of Panitumumab-induced ILD is low the mortality is high.

## Conflicts of interest

No conflicts of interest.
